# Frequency–Time Harmonization of Multi-Station Spectrograms for Machine Learning Applications in Solar Radio Burst Analysis

**DOI:** 10.3390/s26206509

**Published:** 2026-10-14

**Authors:** Vladimir Hristov, Asen Popov, Yuliyan Velchev, Peter Z. Petkov, Christian Monstein

**Affiliations:** 1 Department of Electrical Motion Automation Systems, Faculty of Automatics, Technical University of Sofia, 1000 Sofia, Bulgaria; vdhristov@tu-sofia.bg; 2 Faculty of Computer Systems and Technologies, Technical University of Sofia, 1000 Sofia, Bulgaria; asepopov@tu-sofia.bg; 3 Department of Radio Communications and Video Technology, Faculty of Telecommunications, Technical University of Sofia, 1000 Sofia, Bulgaria; julian_s_velchev@tu-sofia.bg; 4 Istituto Ricerche Solari Aldo e Cele Daccò (IRSOL), Faculty of Informatics, Università della Svizzera Italiana (USI), CH-6605 Locarno, Switzerland; cmonstein@swissonline.ch

**Keywords:** solar radio bursts, spectrogram harmonization, convolutional autoencoders, latent space representation, representation learning, machine learning

## Abstract

The collection of solar radio burst spectrograms through the spatial distribution of survey sites reveals heterogeneity and differences in frequency sampling, temporal resolution, signal intensity, and local observing conditions. Because of this heterogeneity, it is difficult to incorporate observations of diverse stations directly into a single dataset, which restricts the generalizability of machine learning models that were trained using records from isolated stations. Thus, it is well established that for reliable automated analysis of solar radio bursts, a standard representation of spectrograms is necessary. This paper proposes a framework for harmonizing heterogeneous multi-site spectrograms of solar radio bursts, which transforms them into a unified representation capable of accommodating station-specific observational configurations suitable for machine learning applications. The proposed methodology combines adaptive frequency interval selection, frequency sampling consistency checking, logarithmic intensity transformation with baseline correction, time–frequency harmonization, and explicit handling of missing frequency channels using binary validity masks. To overcome the heterogeneous nature of the observations taken from different instruments and stations, nearest-neighbor interpolation in both time and frequency domains is applied. The unified frequency grid is constructed considering the statistics of the frequency channels used in spectrometers that form the e-CALLISTO network, a space weather instrument array. Two one-dimensional convolutional autoencoders with two latent space dimensions (32 and 64) are proposed and trained on more than 17,000 spectrograms to evaluate the quality of latent representations of the harmonized data. The best performance was achieved by the autoencoder with simpler architecture and with 64-dimensional latent space, reaching root-mean-square error of 0.88 dB and a mean absolute error of 0.61 dB. The experimental results show that models with 64-dimensional latent space outperform their 32-dimensional counterparts, while increased architectural complexity provided no substantial improvement. The proposed methodology provides a standardized basis for constructing harmonized datasets suitable for a wide range of subsequent machine learning applications in solar radio astronomy, including automated burst detection, classification, representation learning, anomaly detection, and predictive modeling.

## 1. Introduction

Solar radio bursts are linked to energetic processes occurring in the solar corona and are one of the observed features of particle acceleration in the solar atmosphere [1,2,3]. These emissions are generated by non-thermal electron populations moving through magnetized plasma areas and are tightly coupled to plasma turbulence as well as coronal density variability and magnetic reconnection processes [4,5]. Among the various classes of solar radio bursts, Type III bursts are of particular interest because they are associated with fast-moving electrons propagating along open magnetic field lines into the heliosphere [1,4,5,6,7].

As the electron beams propagate outward through regions with decreasing plasma density, the emitted radio frequency gradually decreases with time [1,4,5]. It is during this process that the characteristic rapidly drifting structures commonly observed on dynamic radio spectrograms are created. The characteristic features of the spectral and temporal evolution of these bursts provide information about electron-beam propagation, plasma conditions, and energy-release mechanisms in the solar atmosphere [3,4,5,6,7]. Dynamic radio spectrograms are therefore commonly employed in both solar physics and space-weather investigations.

At present, continuous monitoring of solar radio activity is carried out using globally distributed radio spectrometer systems operating under different observational conditions. One example is the CALLISTO system and the worldwide e-CALLISTO network, which combines observations from geographically separated radio spectrometers [8,9]. The resulting observations have been widely used for the analysis and visualization of Type II and Type III solar radio bursts [10]. Radio observations of solar activity also provide useful information for space-weather monitoring and related applications [11].

Although distributed observations provide broad temporal and spectral coverage, the resulting spectrogram data remain heterogeneous. Different stations may operate with different frequency coverage, spectral resolution, temporal cadence, receiver sensitivity, channel spacing, and local radio-frequency interference conditions [8,9,10]. Some stations also exhibit irregular or incomplete spectral sampling, making direct comparison and integration of spectrograms from different observational sources more difficult.

Such variability creates considerable difficulties for subsequent machine learning analysis. In recent years, several deep-learning approaches have shown promising performance for automatic solar radio burst detection and classification using spectrogram data [12,13,14,15,16]. However, many of these methods have been developed using station-specific datasets and may therefore remain influenced by the characteristics of the corresponding instruments. In practice, instrumental or station-related characteristics may affect the learned representation if heterogeneous input data are used without appropriate harmonization.

Several preprocessing strategies have been applied to solar radio observations before subsequent analysis or machine-learning processing. These include background subtraction and frequency-range selection [14], normalization and preprocessing of multi-frequency solar radio observations [17], and preprocessing and resampling of spectrogram data before automated classification [18,19]. These approaches demonstrate the importance of reducing instrumental effects and providing a consistent input representation before subsequent analysis.

The present problem additionally involves observations from multiple e-CALLISTO stations with different frequency ranges, channel spacing, temporal resolution, and physically unobserved frequency channels [8,9,10]. A fixed-size representation obtained only by resampling does not explicitly distinguish between measured and unobserved frequency regions. The proposed framework therefore combines conventional preprocessing operations with adaptive frequency-band selection, a common time–frequency grid, and an explicit observation mask. The individual preprocessing operations are not claimed as novel; rather, their integration is intended to provide a consistent representation while retaining information about the actual availability of frequency channels.

Existing studies mainly focus on individual preprocessing steps rather than on the full multi-station harmonization problem addressed in this work. For example, ref. [14] uses background subtraction and frequency-range selection, ref. [17] applies normalization and preprocessing of multi-frequency observations, and refs. [18,19] include preprocessing and resampling as part of classification pipelines. In our case, the problem is broader because the data from different stations may differ at the same time in frequency coverage, channel spacing, temporal resolution, and the availability of measured frequency channels. The proposed framework therefore combines frequency consistency checking, adaptive frequency-band selection, a common time–frequency grid, and an explicit observation mask within one processing procedure. In the reviewed literature, the authors did not find another method that addresses this same combination of multi-station differences; therefore, a direct quantitative comparison with an equivalent harmonization method could not be performed.

In the present work, a harmonization framework for heterogeneous multi-station solar radio spectrograms is proposed. The framework combines logarithmic intensity transformation, baseline correction, frequency consistency validation, and time–frequency harmonization within a common preprocessing procedure. Special attention was given to the stability of the spectral sampling structure because irregular frequency distributions may introduce geometrical distortions during interpolation and may negatively affect subsequent machine learning analysis.

Unlike many station-specific preprocessing procedures, the proposed framework is designed to provide a common representation across heterogeneous observing stations. Although the methodology was validated using observations from the e-CALLISTO network, the proposed harmonization procedure may also be applied to other distributed radio spectrometer systems operating under heterogeneous observational conditions.

The main objective of the proposed framework is to transform spectrograms obtained from different observational stations into a common representation that accommodates differences in station-specific frequency configurations and observational characteristics. The resulting harmonized dataset is intended to provide a standardized basis for machine learning, statistical analysis, anomaly detection, representation learning, and automatic solar radio burst detection.

Finally, the proposed harmonization framework was evaluated using a convolutional autoencoder architecture trained on more than 17,000 harmonized spectrograms obtained from multiple stations. The purpose of this experiment was to evaluate the harmonized representation of heterogeneous multi-station observations and to examine its suitability for the subsequent dimensionality reduction analysis.

The main contributions of this work are

- A reproducible harmonization pipeline that produces a unified time–frequency representation from heterogeneous, station-specific spectrogram data;
- A statistically grounded frequency band selection strategy based on signal visibility and inter-station dispersion;
- A 1D convolutional autoencoder-based representation that compresses the original frequency information into a fixed-size latent vector for each time step, providing a basis for subsequent temporal burst detection and classification.

The presented framework provides a foundation for constructing standardized datasets that can be used in subsequent work for predictive modeling of solar radio bursts.

## 2. Materials and Methods

### 2.1. Overview of the Harmonization Framework

The key goal of the framework proposed in this study is to build a consistent spectrogram dataset that accommodates heterogeneous multi-station solar radio signals. The framework was designed to accommodate differences in instrumental configurations while retaining the physical morphology of solar radio burst structures required for subsequent machine-learning analysis.

The harmonization process includes the spectral matching procedure which consists of spectral validation and frequency–time harmonization. Since the observational data are collected from widely scattered facilities with varying instrumental and environmental conditions, significant fluctuations in raw spectrogram geometry, spectral sampling, temporal cadence, and signal intensity distributions occur. In practice, it is sometimes difficult to compare spectrograms obtained from various stations simply because of these inconsistencies.

The problem is tackled by transforming all spectrograms into a common frequency and time format, and in doing so enabling better processing and analysis. Special attention was given to preserving the stability of the spectral sampling structure, due to inconsistencies with the frequency distribution, which can lead to various geometric distortions during interpolation and lower the performance of the various machine learning methods trained on it.

**Figure 1:**
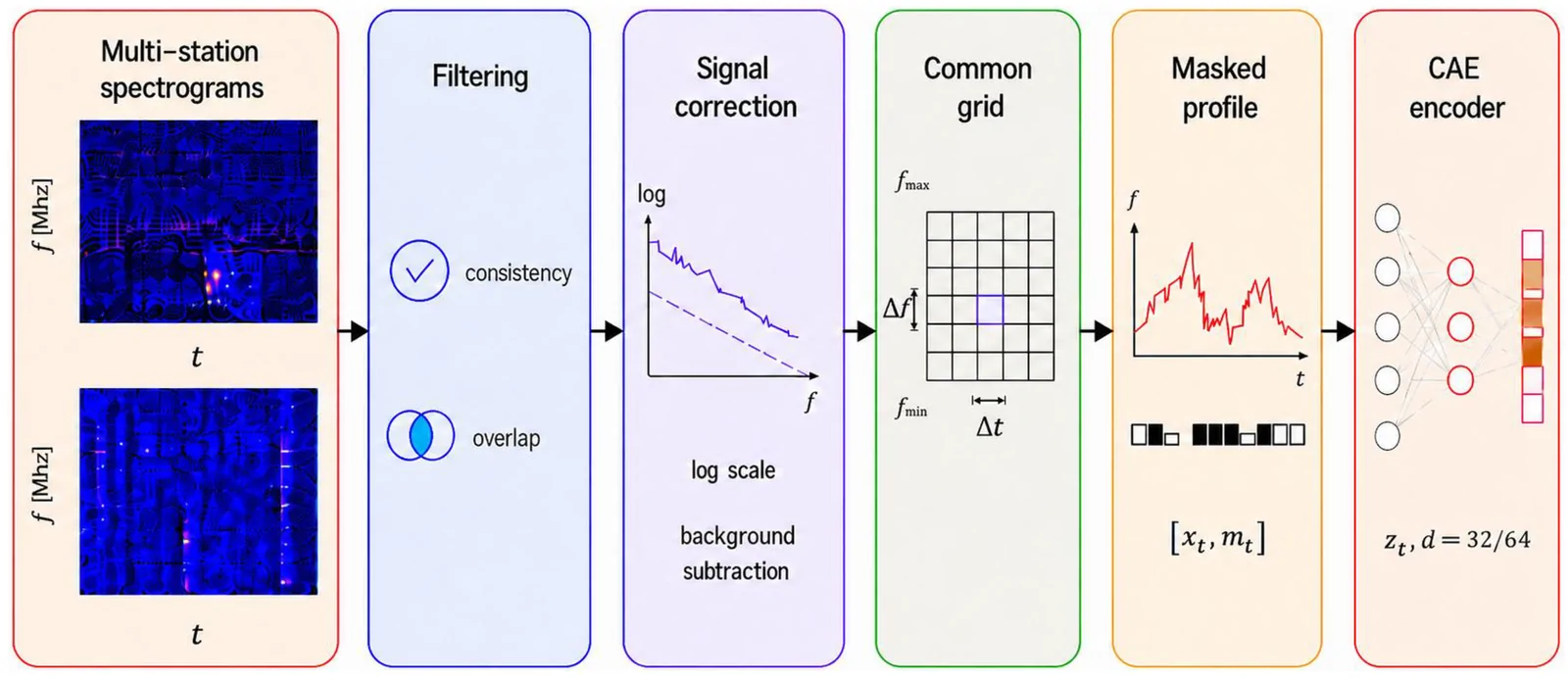
Overview of the proposed preprocessing and representation learning pipeline. Multi-station spectrograms are filtered, corrected, harmonized onto a common frequency–time grid, converted into masked frequency profiles, and encoded by the convolutional autoencoder into fixed-dimensional latent vectors.

Once the harmonization process is complete, the resulting dataset can be used for machine learning, statistical analysis, anomaly detection, and joint analysis of observations from different stations. The entire processing pipeline is shown in Figure 1.

### 2.2. Observational Data

The proposed methodology uses dynamic radio spectrograms collected from globally distributed radio spectrometer stations with heterogeneous observational conditions. In the present study, the framework is validated using observations from the e-CALLISTO network [8,9], with the spectrogram data obtained from the CALLISTO Quicklook archive [20]. Each spectrogram is denoted by X and measures the radio intensity with respect to the frequency *f* and time *t*, where $X\left(f,t\right)$ is the value of X at frequency *f* and time *t*. The observations are obtained from several stations with different instrumental configurations and local environmental conditions, and the observed spectrograms show a considerable diversity among stations due to the highly heterogeneous observing infrastructure. Other variations include changes in frequency coverage, spectral resolution, temporal cadence, receiver sensitivity, channel spacing, and local radio frequency interference conditions.

In addition, some stations work with spectral sampling geometries which are incompletely and irregularly filled, leading to image structure inconsistencies throughout the observational network. This variability does not allow the spectrograms to easily aggregate into a unified dataset, ready for machine learning, and helps determine the requirement for an individual harmonization framework.

### 2.3. Frequency Consistency Validation

The variation in the frequency channel distribution among the observational stations is one of the major problems in multi-station spectrogram harmonization. Some instruments provide nearly uniform frequency sampling, whereas others have irregular, sparse, or partially corrupted sampling patterns. These mismatches can cause geometrical errors in interpolation and could eventually disturb the stability of machine learning models that have been trained on a harmonized dataset. A frequency consistency validation procedure prior to harmonization was implemented to help alleviate this issue.

Let $F=\left\{f_{1},f_{2}\ldots ,f_{n}\right\}$ denote the set of frequency channels associated with a given spectrogram. The spacing between neighboring frequency channels was computed as

(1) $$\Delta f_{i}=f_{i+1}-f_{i}.$$

A representative channel spacing was then estimated using the mean of the spacing distribution $\bar{\Delta f}$. The relative deviation of each frequency interval from the representative spacing was evaluated according to

(2) $$r_{i}=\frac{\left|\Delta f_{i}-\bar{\Delta f}\right|}{\bar{\Delta f}}.$$

The filtered dataset was subsequently defined as

(3) $$D_{\mathrm{filtered}}=\left\{X_{k}\in D|M_{k}\leq \tau \right\},$$

where $M_{k}=\mathrm{mean}\left(r_{i}\right)$ for the *k*-th spectrogram, τ=0.1 is the tolerance threshold that specifies the maximum allowed frequency irregularity, and D is the original dataset.

The tolerance threshold was chosen empirically for the current study in view of the stability of the spectral sampling distributions observed. Spectrograms with high levels of irregularity were no longer included in the harmonized set. In this validation phase only observations with stable spectral geometries were kept as precursors for further interpolation and normalization procedures.

### 2.4. Frequency Interval Selection

Since the spectral ranges of different observational stations partially overlap, a common harmonized frequency interval must be selected before interpolation and resampling. The developed methodology determines the harmonized interval dynamically based on the common spectral overlap and frequency consistency statistics of the participating stations rather than establishing a fixed frequency interval in the context of a defined observational network. The harmonized frequency interval $I^{*}=\left[f_{\min},f_{\max}\right]$ should overlap with at least a fraction $p_{\min}$ of the spectrograms in $D_{\mathrm{filtered}}$. The problem can be formulated as

(4) $$|\left\{I_{k}|I_{k}\cap I^{*}\neq \emptyset \right\}|\geq p_{\min}\;\cdot \;N,$$

where $I_{k}=\left[f_{k,\min},f_{k,\max}\right]$ is the frequency interval for the *k*-th spectrogram in $D_{\mathrm{filtered}}$, and *N* is the number of elements in $D_{\mathrm{filtered}}$. In the present work, the value $p_{\min}=0.33$ was used, meaning that at least 33% of the spectrograms must contain the corresponding frequency region. The sweep-line (event-sorting) approach is employed to solve the problem. For each observation (spectrogram), two events are defined. The first is the beginning of the interval ($f_{k,\min}\to +1$) and the second is the end of the interval ($f_{k,\max}\to -1$). A sorted list of frequency events $\left(f,\pm 1\right)$ is produced as a result. The events are traversed in ascending order of frequency while maintaining the current number of active intervals $C\left(f\right)$. If $C\left(f\right)\geq p_{\min}\;\cdot \;N$, the frequency interval is considered valid. The widest interval among all valid intervals is selected according to

(5) $$I^{*}=\arg\max_{I}\left(f_{\max}-f_{\min}\right).$$

According to the given parameters, the resulting appropriate frequency range spans from $f_{\min}=15\;\mathrm{MHz}$ to $f_{\max}=95\;\mathrm{MHz}$.

Finally, each spectrogram is evaluated according to the overlap between its individual frequency interval and the determined global interval. For further processing, only spectrograms with at least 20% overlap with $I^{*}$ are retained. The described procedure ensures maximal utilization of the available spectrograms while maintaining a consistent frequency-domain representation across the resulting dataset.

The proposed procedure was applied to the e-CALLISTO dataset covering the period from 2020 to the end of 2024, considering only spectrograms containing identified events (solar bursts). Since the events typically exhibit relatively short temporal durations, this filtering does not significantly reduce the generality of the resulting dataset, as the majority of time steps still correspond to non-event regions. Starting from more than 6,650,000 spectrograms, the final dataset was reduced to 24,875 after all preprocessing and filtering stages.

### 2.5. Logarithmic Scaling and Baseline Correction

The raw spectrogram intensities exhibit large dynamic ranges and strong station-dependent amplitude variability. To stabilize the signal distribution and reduce the influence of extreme intensity variations, the spectrogram intensities are transformed into logarithmic space:

(6) $$X_{\mathrm{dB}}\left(f,t\right)=\frac{X\left(f,t\right)}{255}\frac{2500}{25.4}\approx 0.386X\left(f,t\right),$$

where 255 is the maximal code from the analog-to-digital converter, 2500 mV corresponds to the voltage range, and 25.4 $\mathrm{mV}\;{\mathrm{dB}}^{-1}$ is the detector sensitivity. The same transformation is used for the visualisation of spectrograms in the e-CALLISTO Quicklook archive [20].

Following the logarithmic transformation, baseline correction is applied independently to each frequency channel to suppress stationary instrumental background components. The baseline is estimated using the temporal median intensity. The median provides a robust estimate of the background level and is less sensitive to short-duration intensity variations than the arithmetic mean [21]:

(7) $$B\left(f\right)={\mathrm{median}}_{t}\left(X_{\mathrm{dB}}\left(f,t\right)\right).$$

The corrected spectrogram is

(8) $$X_{\mathrm{dB}}^{\prime }\left(f,t\right)=X_{\mathrm{dB}}\left(f,t\right)-B\left(f\right).$$

### 2.6. Time–Frequency Harmonization

After spectral validation and adaptive frequency band selection, all spectrograms are mapped onto a unified frequency–time grid. Temporal resampling is additionally applied to obtain a consistent time-axis representation across observations originating from different stations. Such resampling provides a consistent discrete-time representation of signals acquired with different temporal sampling characteristics [22]. The spectrograms are subsequently resampled onto the unified frequency–time grid using nearest-neighbor interpolation. The fixed frequency resolution is 250 kHz, while the time step is 250 ms.

Because the participating stations do not all observe the same frequency channels, the unified grid may contain frequency bins that are not covered by a given station. After interpolation, such uncovered entries are represented by NaN values and are encoded as a binary observation mask m associated with each harmonized spectrogram X. Let $m\left(f,t\right)=1$ denote an observed value at frequency *f* and time *t*, and $m\left(f,t\right)=0$ denote a missing one. The numerical spectrogram values are obtained by replacing the missing entries with zero, while the mask m retains the information about which frequency bins are physically observed. This observation mask is propagated together with the harmonized spectrogram through the subsequent representation learning and evaluation stages. Thus, unobserved frequency channels remain explicitly distinguishable from physically measured values throughout the subsequent representation-learning procedure.

### 2.7. Masked One-Dimensional Convolutional Autoencoder

The harmonized spectrogram representation is further compressed using a mask-aware one-dimensional convolutional autoencoder [23]. After preprocessing and interpolation onto the common frequency grid, each time step of a spectrogram is treated as an individual frequency profile. For a spectrogram with *F* frequency bins and *T* time samples, the autoencoder input at time index *t* is therefore a vector $x_{t}\in R^{F}$. This formulation preserves the temporal ordering externally while allowing the encoder to learn compact frequency-domain representations for each time step independently.

The binary observation mask m introduced in Section 2.6 is provided to the network as an additional input channel alongside the measured intensity profile. For a given time step *t*, the corresponding mask column $m_{t}$ has entries $m_{t,i}\in \left\{0,1\right\}$ indicating whether frequency bin *i* is physically observed. The network therefore receives both the measured intensity profile and the information about which frequency bins are observed.

The autoencoder consists of an encoder $E_{\theta }$ and a decoder $D_{\phi }$. The encoder maps each masked frequency profile to a compact latent representation,

(9) $$z_{t}=E_{\theta }\left(x_{t},m_{t}\right),$$

where $z_{t}\in R^{d}$ and *d* is the latent space dimension. The decoder reconstructs the original frequency profile from this latent vector,

(10) $${\hat{x}}_{t}=D_{\phi }\left(z_{t}\right).$$

Training is performed using a masked mean-squared reconstruction loss,

(11) $$L_{\mathrm{CAE}}=\frac{\sum_{t=1}^{T}\sum_{i=1}^{F}m_{t,i}{\left(x_{t,i}-{\hat{x}}_{t,i}\right)}^{2}}{\sum_{t=1}^{T}\sum_{i=1}^{F}m_{t,i}},$$

so missing frequency bins do not contribute to the optimization objective. This is important because the goal is not to force the model to reproduce unavailable measurements, but to evaluate whether the observed parts of the harmonized spectrogram can be represented consistently in a compact latent space.

**Figure 2:**
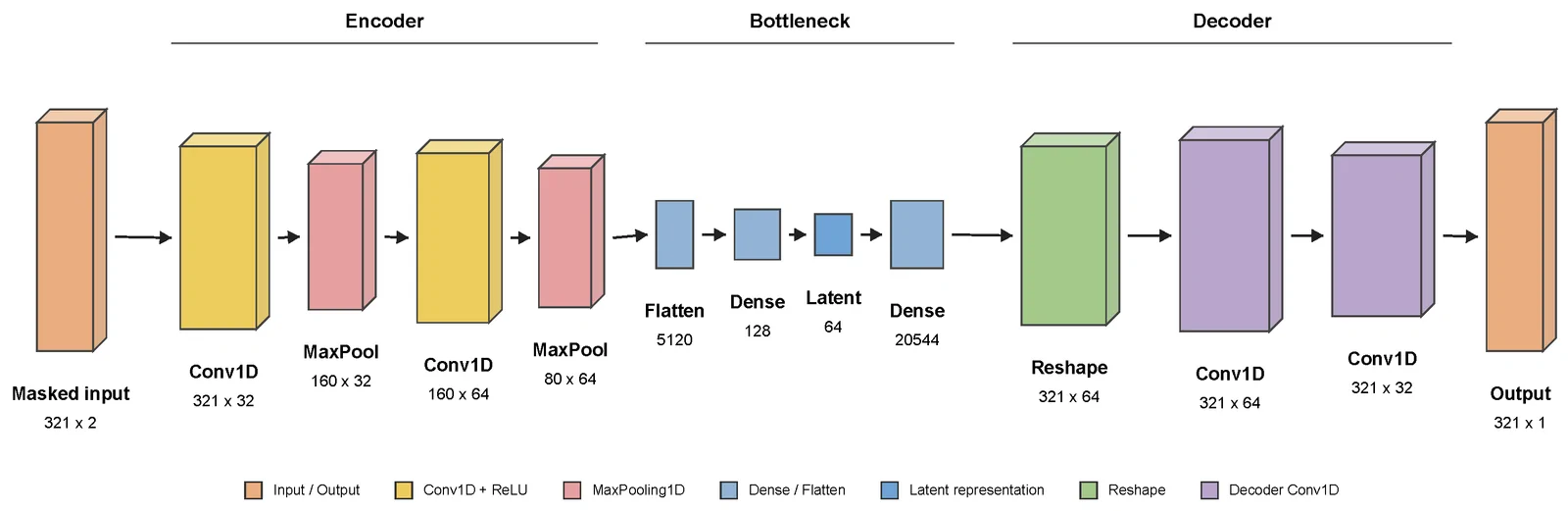
Baseline masked one-dimensional convolutional autoencoder architecture. Each time step is represented as a frequency profile with an additional observation-mask channel. The encoder compresses the profile into a low-dimensional latent vector, and the decoder reconstructs the corresponding frequency profile.

**Figure 3:**
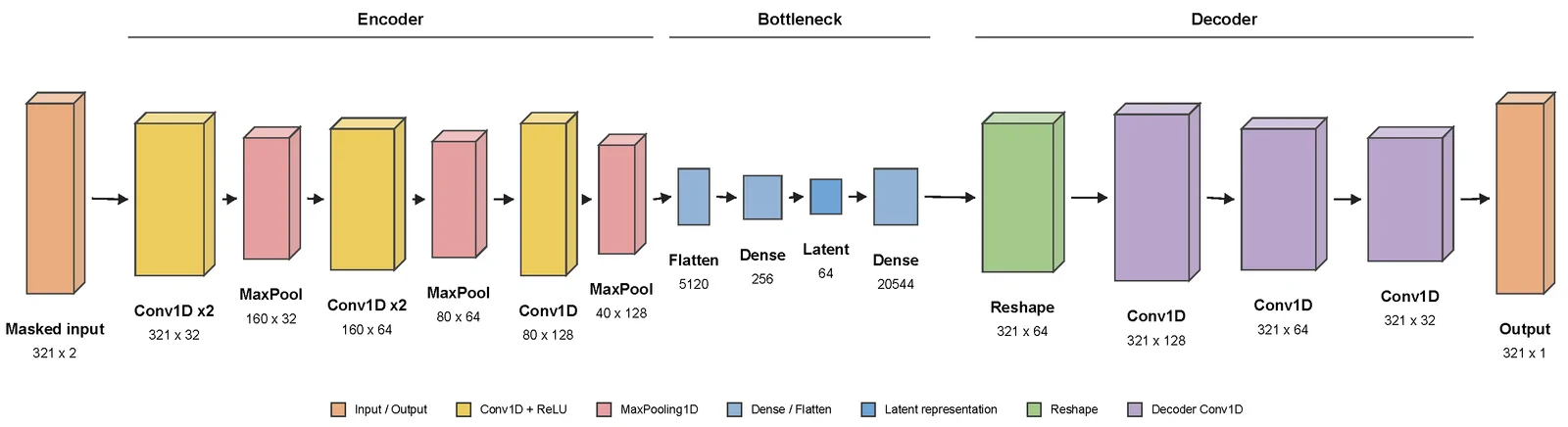
Deeper masked one-dimensional convolutional autoencoder architecture. Compared with the baseline model, this variant uses additional convolutional processing before the latent representation in order to test whether increased architectural capacity improves masked spectrogram reconstruction.

Two convolutional autoencoder variants were investigated. The baseline masked one-dimensional CAE uses a compact encoder–decoder structure with two convolutional stages followed by dense latent compression, as shown in Figure 2. A deeper variant, shown in Figure 3, increases the number of convolutional layers and introduces an additional convolutional block before the latent projection. Both variants use one-dimensional convolutions along the frequency axis [24] and reconstruct one frequency profile at a time.

### 2.8. Implementation Details

The complete harmonization and representation learning pipeline was implemented in Python (version 3.11.7) using standard scientific computing and deep learning libraries. The preprocessing stage comprises frequency consistency verification, adaptive frequency interval selection, interpolation onto the common frequency–time grid, construction of the binary observation mask, logarithmic scaling, and per-channel baseline correction, applied uniformly to observations collected from different stations. The masked one-dimensional convolutional autoencoder was implemented and trained within the same framework. Fixed random seeds were used throughout to ensure reproducibility of the dataset splitting and model initialization. The harmonized representation and the trained autoencoder models together provide a reusable basis for the experiments reported in the following section, where the consistency of the harmonized spectrogram representations is assessed through convolutional autoencoder reconstruction across heterogeneous multi-station observations.

## 3. Results

### 3.1. Model Training

This section presents the experimental evaluation of the proposed harmonization framework. The quality of the harmonized spectrogram representation is assessed through reconstruction experiments using the masked convolutional autoencoder described in Section 2.7. The autoencoder training set was constructed from the harmonized spectrogram representation as described in Section 2.4. The 24,875 spectrograms available for training and testing correspond to approximately 89,550,000 samples, considering the typical spectrogram duration of about 15 min and selected time step of 250 ms.

To avoid information leakage between neighboring time samples from the same observation, the data were split at the file level before extracting the final training samples. Entire spectrogram files were assigned to the training, validation, or test partitions, and only then were the time-step profiles generated. The final split used 70% of files for training, 20% for validation, and 10% for testing, which corresponds to more than 17,000 spectrograms in the training partition and the remaining files distributed between validation and testing. Because each file expands into approximately 3600 frequency-profile samples, this file-level partitioning yields a training set on the order of tens of millions of profiles while ensuring that adjacent columns from the same spectrogram do not appear in different data partitions. Validation data were used for model selection and early stopping, while held-out test files were reserved for final reconstruction assessment.

The input values were normalized after background subtraction and scaled to a bounded numerical range before being passed to the network. Missing values were preserved as NaN until batch construction; they were then replaced by zeros only in the numerical input channel, while the corresponding mask channel indicated which frequency bins were physically observed. The same masked reconstruction loss in Equation (11) was used for all model variants, so only observed bins contributed to the optimization objective.

Four controlled autoencoder configurations were trained by combining the two architecture variants with two latent space dimensions, 32 and 64. The baseline and deeper models were trained with the same preprocessing settings, optimizer type, validation strategy, and reconstruction objective, allowing the effect of latent dimensionality and architectural complexity to be evaluated separately. All models were optimized using Adam with a learning rate of 1 × 10^−3^, a batch size of 256, and a maximum of 10 training epochs. The random seed was fixed to 42 for reproducible file splitting and model initialization. Early stopping was applied using the validation reconstruction loss with a patience of 3 epochs, and the best validation weights were restored before test evaluation. Reconstruction quality was assessed using masked error metrics computed only over observed frequency bins, including masked root-mean-square error and mean absolute error in dB space.

The latent representation produced by the encoder is intended to provide a fixed-dimensional description of each time step, independent of the number of observed frequency bins in the original station data. This makes the representation suitable for downstream time-step-level analysis, including future Type III solar radio burst detection or classification models operating on latent sequences rather than directly on heterogeneous spectrogram grids.

**Figure 4:**
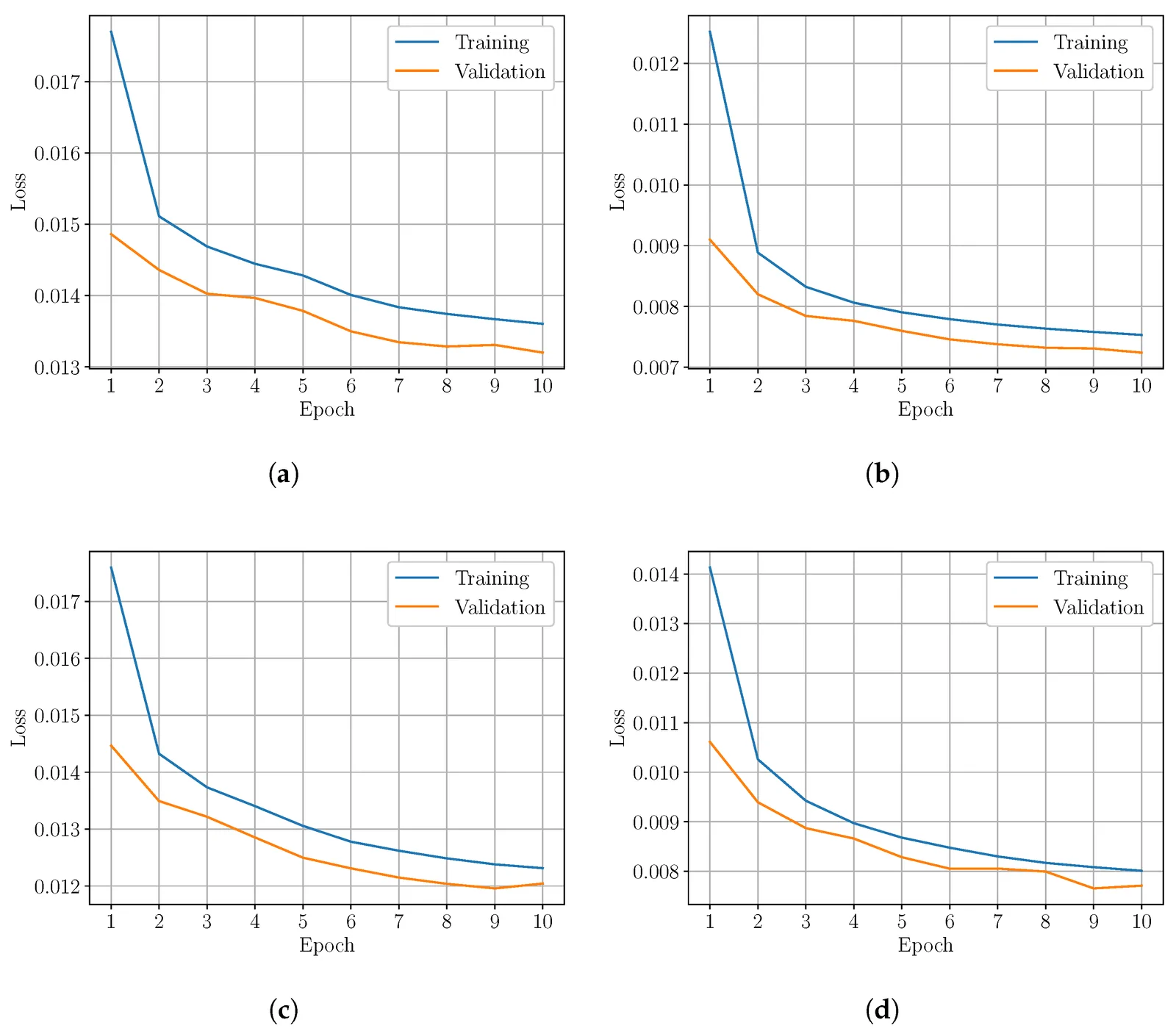
Training and validation reconstruction loss as a function of the training epoch for the four model configurations: (**a**) baseline model with 32-dimensional latent space, (**b**) baseline model with 64-dimensional latent space, (**c**) deeper model with 32-dimensional latent space, and (**d**) deeper model with 64-dimensional latent space.

Figure 4 shows the evolution of the training and validation reconstruction loss over the ten training epochs for the four model configurations.

In all cases, both curves decrease rapidly during the first two to three epochs and then continue to decline more gradually, flattening towards the end of training. The validation loss closely follows the training loss throughout, and the 64-dimensional models (panels (b) and (d)) reach markedly lower loss values than their 32-dimensional counterparts (panels (a) and (c)), in agreement with the quantitative metrics reported below.

The chosen budget of 10 training epochs is adequate for the present case. Convergence is clearly visible, as the loss curves have flattened by the final epochs, and the validation loss exhibits no upward jump or systematic divergence from the training loss. The absence of such a gap indicates that the models do not overfit within the considered training horizon, so a longer training schedule is not required.

### 3.2. Reconstruction Performance

The reconstruction performance was evaluated in the logarithmic domain using masked Root Mean Squared Error (RMSE) and Mean Absolute Error (MAE). Each spectrogram time step was regarded as an independent one-dimensional observation over the frequency axis. Let $x_{t,f}$ denote the ground-truth spectrogram magnitude at time step *t* and frequency bin *f*, ${\hat{x}}_{t,f}$ denote the reconstructed value, and $m_{t,f}\in \left\{0,1\right\}$ represent a binary mask indicating valid frequency entries. The evaluation metrics were computed only over valid (unmasked) elements:

(12) $${\mathrm{RMSE}}_{\mathrm{masked}}=\sqrt{\frac{\sum_{t=1}^{T}\sum_{f=1}^{F}m_{t,f}{\left(x_{t,f}-{\hat{x}}_{t,f}\right)}^{2}}{\sum_{t=1}^{T}\sum_{f=1}^{F}m_{t,f}}}$$

and

(13) $${\mathrm{MAE}}_{\mathrm{masked}}=\frac{\sum_{t=1}^{T}\sum_{f=1}^{F}m_{t,f}\left|x_{t,f}-{\hat{x}}_{t,f}\right|}{\sum_{t=1}^{T}\sum_{f=1}^{F}m_{t,f}},$$

where *T* denotes the total number of time samples and *F* is the number of frequency bins. The achieved results for 32- and 64-dimensional latent space for baseline and deeper models are shown in Table 1.

The test loss values obtained for the baseline and deeper models with different latent space dimensions are summarized in Table 2.

**Figure 5:**
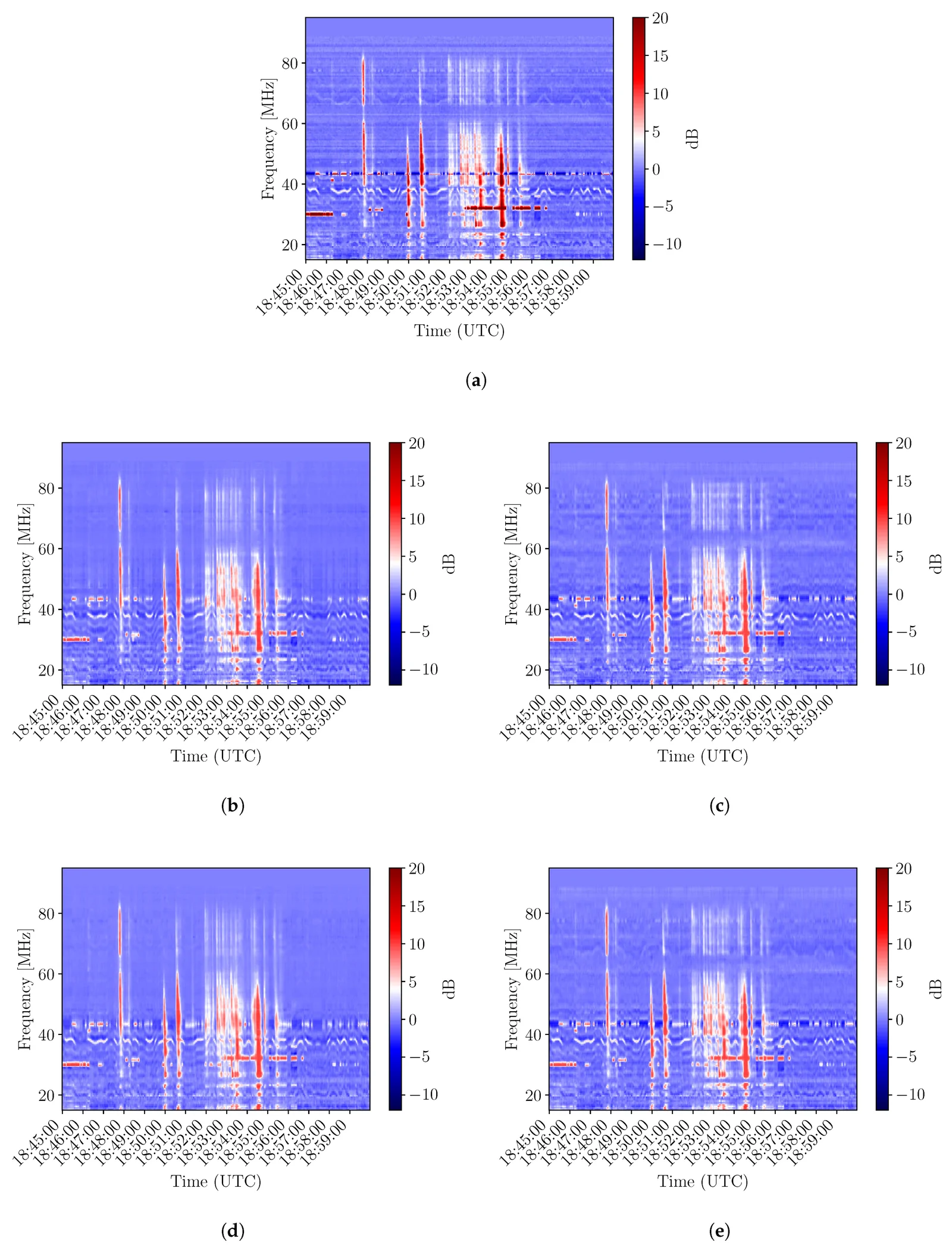
Example spectrogram reconstructions from the ALASKA-COHOE instrument, part of the e-CALLISTO network, on 22 July 2024: (**a**) Original preprocessed spectrogram, (**b**) reconstructed using the baseline model with 32-dimensional latent space, (**c**) reconstructed using the baseline model with 64-dimensional latent space, (**d**) reconstructed using the deeper model with 32-dimensional latent space, and (**e**) reconstructed using the deeper model with 64-dimensional latent space.

Example spectrogram reconstructions from the ALASKA-COHOE instrument, part of the e-CALLISTO network, are shown in Figure 5.

The given results indicate that increasing the latent space dimensionality improves reconstruction performance for both baseline and deeper architectures. The 64-dimensional baseline model achieved the best performance, obtaining the lowest masked RMSE and MAE values. While the deeper architecture provided slightly better performance than the baseline model for the 32-dimensional latent space, this advantage did not persist for the 64-dimensional configuration, indicating that latent space dimensionality has a stronger influence on reconstruction performance than architectural complexity. Visual inspection of the reconstructed spectrograms confirms that the baseline model with a 64-dimensional latent space provides the most accurate reconstruction. Increased reconstruction errors are observed in regions with finer details, as can be expected for autoencoder-based models.

Overall, the quantitative metrics reported in Table 1 and Table 2 together with the qualitative reconstruction examples shown in Figure 5 indicate that the proposed framework preserves the dominant morphology of solar radio bursts while enabling compact latent representations across heterogeneous multi-station observations.

### 3.3. Comparison with Incremental Principal Component Analysis

To provide a conventional linear baseline for the compact representations obtained with the autoencoder, Incremental Principal Component Analysis (IPCA) was additionally evaluated [25]. The incremental implementation permits the large number of frequency profiles in the dataset to be processed in mini-batches. Each original harmonized frequency profile has a dimensionality of 321 elements, whereas the autoencoder models considered in this study reduce it to 32 or 64 latent dimensions, corresponding to approximately 10.0% and 19.9% of the original dimensionality, respectively.

IPCA requires a complete numerical input matrix and cannot use the observation mask during model fitting. Therefore, missing entries were imputed with the corresponding median value. The IPCA experiments used the same training and test sets as the autoencoder experiments to ensure a direct comparison. IPCA was evaluated with 16, 32, 48, 64, 80, 96, 112, and 128 principal components. Reconstructed profiles were evaluated using the same masked RMSE and MAE definitions in (12) and (13), so imputed, physically unobserved entries did not contribute to the reported errors. Cumulative explained variance was also calculated to quantify the proportion of data variability retained by each linear representation.

**Figure 6:**
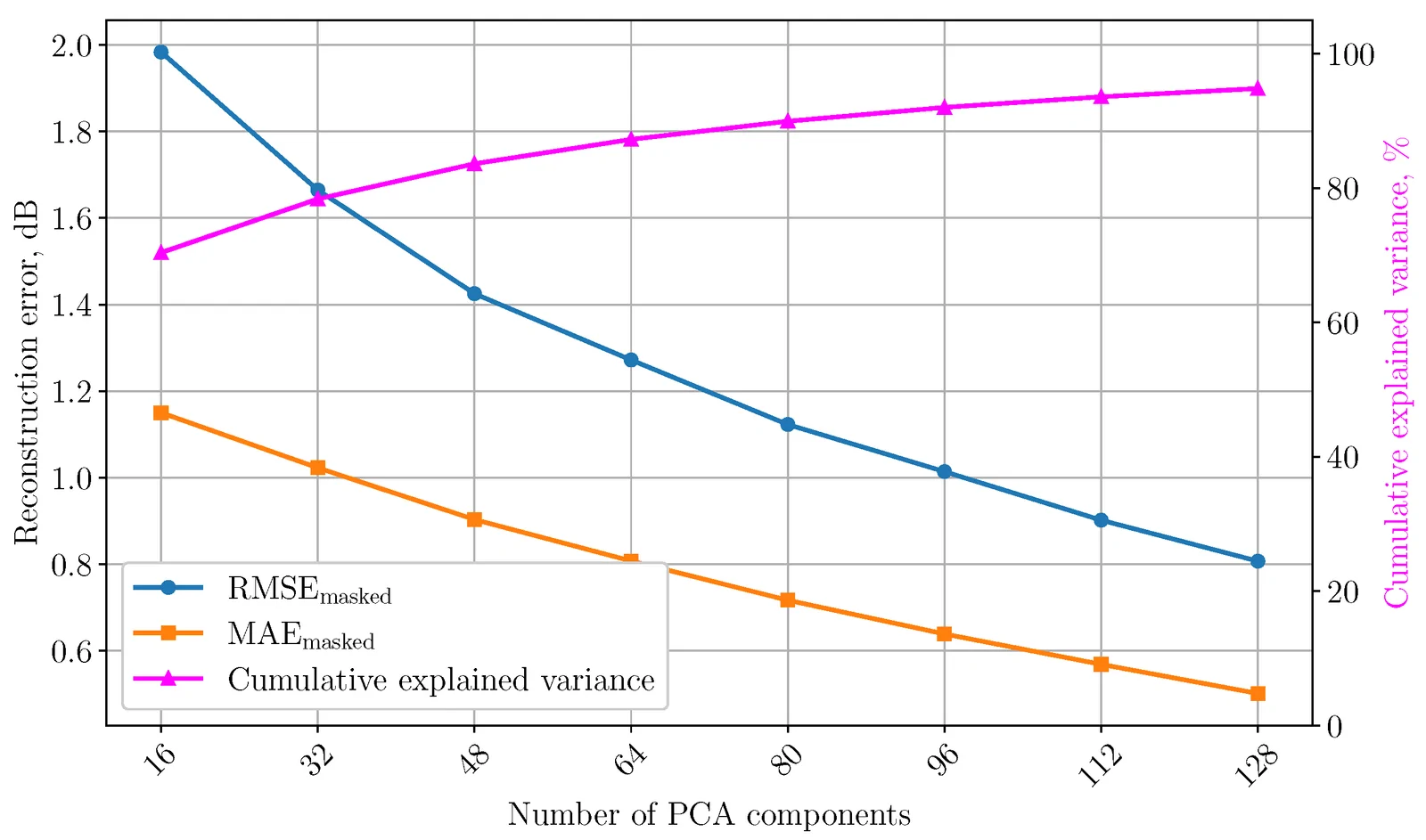
Incremental Principal Component Analysis reconstruction performance for different numbers of retained principal components. The figure shows masked RMSE, masked MAE, and cumulative explained variance.

As shown in Figure 6, the reconstruction error decreases and the cumulative explained variance increases as more principal components are retained. IPCA retains approximately 88% of the variance with 64 components and approximately 95% with 128 components.

With 64 components, IPCA obtains a masked RMSE of approximately 1.27 dB and a masked MAE of approximately 0.81 dB, whereas the baseline masked CAE obtains 0.877 dB and 0.612 dB. Thus, the autoencoder yields lower reconstruction error with the matched 64-dimensional representation. IPCA approaches the reconstruction accuracy of the 64-dimensional CAE only when the number of retained components is increased to approximately 112–128. In contrast, the 64-dimensional CAE reduces the original 321-dimensional frequency profile by approximately 80%. These results indicate that the nonlinear autoencoder preserves reconstruction-relevant structure more compactly than the linear baseline. They also reinforce the practical advantage of the mask-aware CAE for the present heterogeneous dataset: unlike IPCA, it incorporates frequency-channel availability directly and does not require median imputation of missing measurements.

## 4. Discussion

The results show that the proposed harmonization framework transforms heterogeneous observations acquired from different stations into a common time–frequency representation while preserving the dominant morphology of solar radio bursts. By combining preprocessing and time–frequency harmonization within a common procedure, observations obtained from instruments with different frequency configurations can be represented in a consistent format suitable for subsequent machine-learning analysis.

The comparison between the 32- and 64-dimensional latent spaces indicates that latent dimensionality has a clear effect on reconstruction quality. Both architecture variants improved when the latent space was increased to 64 elements, suggesting that the lower-dimensional representation was too restrictive for preserving the relevant frequency-domain structure. The best reconstruction performance was obtained by the baseline model with a 64-dimensional latent space, which achieved the lowest masked RMSE, MAE, and test loss values in Table 1 and Table 2.

The deeper architecture did not provide a consistent advantage over the baseline model. Although it slightly improved performance for the 32-dimensional latent space, this improvement did not persist for the 64-dimensional configuration. This suggests that, for the current representation and dataset, the bottleneck capacity is more important than increasing convolutional depth. A simpler model may therefore be preferable, since it is easier to train, easier to interpret, and more suitable as a preprocessing stage for downstream temporal models.

The comparison with IPCA provides additional context for these results. At the same representation dimensionality, the nonlinear autoencoder achieves lower reconstruction error than the linear baseline. Considering that the original frequency profile contains 321 elements, the 64-dimensional latent representation reduces the dimensionality by approximately 80%. IPCA requires a larger number of components to approach similar reconstruction accuracy.

The reconstruction examples further support this interpretation. The main spectrogram structure is preserved by the trained models, while larger errors remain in regions with fine temporal or spectral detail. This behavior is expected for autoencoder-based compression: the model captures the dominant structure of the frequency profile, but very localized or low-amplitude features may be smoothed by the latent bottleneck. For the intended use as a representation-learning stage, this trade-off is acceptable if the latent sequence retains the information needed for later burst detection or temporal classification.

The present evaluation also has several limitations. Although the dataset contains observations from multiple stations, the current file-level split does not constitute a strict leave-one-station-out evaluation. Therefore, generalization to completely unseen station configurations remains to be evaluated separately. In addition, a systematic ablation of the observation mask, baseline correction, adaptive frequency band selection, and interpolation strategy would help quantify the individual contribution of each preprocessing component.

Future research will focus on applying the proposed harmonization framework to the automatic detection and classification of radio bursts, self-supervised representation learning, anomaly detection, and the prediction of solar radio activity.

## 5. Conclusions

This work presents a harmonization framework for multi-station e-CALLISTO solar radio spectrograms and demonstrates its use for training masked one-dimensional convolutional autoencoders. The framework standardizes heterogeneous observations, preserves missing frequency channels through explicit masks, and produces fixed-dimensional latent representations for each spectrogram time step.

The comparison with IPCA further showed that the nonlinear autoencoder achieves lower reconstruction error at the same representation dimensionality while reducing the original 321-dimensional frequency profile to a compact 64-dimensional representation.

The experimental results indicate that a baseline CAE with a 64-dimensional latent space provides the best reconstruction performance among the tested configurations. This supports the use of a lightweight mask-aware autoencoder as a representation-learning stage before downstream solar radio burst detection.

Future work will use the resulting latent sequences as observations for a Hidden Markov Model (HMM), with the goal of modeling temporal transitions between quiet background, burst onset, burst body, and decay phases. Additional work will include evaluation on larger multi-year datasets, integration of manually verified Type III annotations, comparison with alternative temporal models, and investigation of attention-based or Transformer-style architectures for capturing broader time–frequency context.

## Figures and Tables

**Table 1:** Reconstruction performance evaluation metrics of baseline and deeper models, achieved with different latent space dimensions.

Model Type	Latent Dimension	${\mathrm{RMSE}}_{\mathrm{masked}}$, dB	${\mathrm{MAE}}_{\mathrm{masked}}$, dB
Baseline	32	1.178	0.815
Baseline	64	0.877	0.612
Deeper	32	1.121	0.777
Deeper	64	0.901	0.628

**Table 2:** Test loss achieved by baseline and deeper models for different latent space dimensions.

Model Type	Latent Dimension	Test Loss
Baseline	32	0.01366
Baseline	64	0.00755
Deeper	32	0.01237
Deeper	64	0.00797

## Data Availability

The dataset used for experimental purposes in this study is publicly available at https://soleil.i4ds.ch/solarradio/callistoQuicklooks/ (accessed on 6 October 2026). The source codes are available from the corresponding author upon reasonable request.

## Abbreviations

The following abbreviations are used in this manuscript:

1D	One-dimensional
CAE	Convolutional autoencoder
FITS	Flexible Image Transport System
HMM	Hidden Markov Model
IPCA	Incremental principal component analysis
LOFAR	Low-Frequency Array
MAE	Mean absolute error
RMSE	Root-mean-square error
